# Monitoring arthropods under the scope of LIFE-SNAILS project: I - Santa Maria Island baseline data with implementation of the Index of Biotic Integrity

**DOI:** 10.3897/BDJ.12.e116829

**Published:** 2024-02-23

**Authors:** Paulo A. V. Borges, Lucas Lamelas-López, Sébastien Lhoumeau, Nelson B. Moura, Mauro Ponte, Abrão Leite, Laurine Parmentier, Ricardo Abreu

**Affiliations:** 1 cE3c- Centre for Ecology, Evolution and Environmental Changes/Azorean Biodiversity Group, CHANGE – Global Change and Sustainability Institute, School of Agricultural and Environmental Sciences, University of the Azores, Rua Capitão João d´Ávila, Pico da Urze, 9700-042, Angra do Heroísmo, Azores, Portugal cE3c- Centre for Ecology, Evolution and Environmental Changes/Azorean Biodiversity Group, CHANGE – Global Change and Sustainability Institute, School of Agricultural and Environmental Sciences, University of the Azores, Rua Capitão João d´Ávila, Pico da Urze, 9700-042 Angra do Heroísmo, Azores Portugal; 2 IUCN SSC Atlantic Islands Specialist Group, 9700-042, Angra do Heroísmo, Azores, Portugal IUCN SSC Atlantic Islands Specialist Group, 9700-042 Angra do Heroísmo, Azores Portugal; 3 IUCN SSC Species Monitoring Specialist Group, 9700-042, Angra do Heroísmo, Azores, Portugal IUCN SSC Species Monitoring Specialist Group, 9700-042 Angra do Heroísmo, Azores Portugal; 4 Secretaria Regional do Ambiente e Alterações Climáticas, Project LIFE SNAILS (LIFE20 NAT/PT/001377), Rua Dr. Teófilo Braga nº 10/12/14, 9580 – 535, Vila do Porto, Santa Maria, Azores, Portugal Secretaria Regional do Ambiente e Alterações Climáticas, Project LIFE SNAILS (LIFE20 NAT/PT/001377), Rua Dr. Teófilo Braga nº 10/12/14, 9580 – 535 Vila do Porto, Santa Maria, Azores Portugal; 5 Secretaria Regional do Ambiente e Alterações Climáticas, Project LIFE SNAILS (LIFE20 NAT/PT/001377), Rua do Galo nº 118, 9700-040, Angra do Heroísmo, Terceira, Azores, Portugal Secretaria Regional do Ambiente e Alterações Climáticas, Project LIFE SNAILS (LIFE20 NAT/PT/001377), Rua do Galo nº 118, 9700-040 Angra do Heroísmo, Terceira, Azores Portugal; 6 Rua Fernando Pessoa, nº99 R/C DTO 2765-483, Estoril, Portugal Rua Fernando Pessoa, nº99 R/C DTO 2765-483 Estoril Portugal; 7 Rua da Oliveira nº8, 9700-136 Sé, Angra do Heroísmo, Azores, Portugal Rua da Oliveira nº8, 9700-136 Sé Angra do Heroísmo, Azores Portugal

**Keywords:** arthropods, Azores, Index of Biotic Integrity (IBI), long-term monitoring, Macaronesia, SLAM traps

## Abstract

**Background:**

The database we introduce is a pivotal component of the LIFE SNAILS project (Support and Naturalisation in Areas of Importance for Land Snails). This initiative is dedicated to safeguarding three endangered species of terrestrial molluscs, specifically, two snails (*Oxychilusagostinhoi* Martins 1981 and *Leptaxisminor* Backhuys, 1975) and a semi-slug (*Plutoniaangulosa* (Morelet, 1860)), all of which are single island endemics from Santa Maria Island and face significant threats towards their populations.

In this study, we established a comprehensive database derived from a long-term arthropod monitoring campaign utilising SLAM (Sea, Land, Air, Malaise) traps. Although molluscs were not the primary focus, our findings serve as a credible proxy for evaluating the overall habitat quality for endemic invertebrates, with arthropods serving as principal indicators. From September to December of 2022, a total of 11 SLAM traps were installed and monitored monthly in eleven sites of mixed forests of Santa Maria Island.

**New information:**

Based on the 33 available samples (11 sites x 3 sampling periods), we recorded a total of 118 taxa of arthropods (of which 94 were identified at species or subspecies level), belonging to three classes, 14 orders and 62 families. From the 94 identified taxa, a total of 21 species were endemic, 31 native non-endemic, 32 introduced and 10 indeterminate. We also provide additional information of the habitat quality (Index of Biotic Integrity), including general habitat and dominant species composition.

We registered three new records to the Island, the native bug *Piezodoruslituratus* (Fabricius, 1794) (Hemiptera, Pentatomidae), the Azorean endemic beetle *Phloeosinusgillerforsi* Bright, 1987 (Coleoptera, Curculionidae) and the exotic ant *Hypoponerapunctatissima* (Roger, 1859) (Hymenoptera, Formicidae) and one new record for the Azores Archipelago, the native beetle *Cephenniumvalidum* Assing & Meybohm, 2021 (Coleoptera, Staphylinidae, Scydmaeninae).

This publication not only contributes to the conservation of highly threatened endemic molluscs, through an assessment of habitat quality, based on arthropod communities and habitat description (e.g. native or exotic vegetation), but also provides an updated inventory of arthropods from Santa Maria Island.

## Introduction

Landscape transformation, particularly through the replacement of natural habitats with agricultural areas, stands out as a primary contributor to global biodiversity loss (Diamond et al. 1997, Arnillas et al. 2017, Ntshanga et al. 2021, Ramos et al. 2022). The effects of landscape transformation are especially dramatic in island ecosystems, because despite encompassing merely about 5% of the world's land surface, islands are recognised as biodiversity hotspots (Myers et al. 2000), harbouring threatened (Kier et al. 2009, Tershy et al. 2015) and endemic (Myers et al. 2000, Kier et al. 2009) species. Additionally, population decline and/or species extinction are disproportionately rapid on islands (Tershy et al. 2015). Therefore, islands are an epicentre of biodiversity loss (Spatz et al. 2017, Borges et al. 2019).

In particular, the Azorean landscape has suffered severe transformations since Portuguese colonisation in the 15^th^ century, mainly associated with the replacement of native forests by agricultural fields, forestry plantations and urban areas (Gaspar et al. 2008, Borges et al. 2019, Tsafack et al. 2023b). Less than 3% of the Archipelago land surface is currently covered by pristine forest (Gaspar et al. 2008). These pristine forests are currently under severe threat from invasive plant species and associated habitat degradation (Borges et al. 2019).

The concept of biotic integrity is often associated with the absence of external human influence, using pristine sites as benchmarks (Margules et al. 1994, Cardoso et al. 2006). To assess a site's biological integrity, comparisons are made with these benchmarks, based on defined criteria (Cardoso et al. 2006). Surrogate measures like habitat quality indices provide quick assessments of integrity by incorporating rough measures of habitat disturbance, mainly related with human activities (Angermeier and Davideanu 2004, Cardoso et al. 2006). Recent studies, such as Tsafack et al. (2023b), have developed biological integrity indices using the arthropod communities as indicators to assess the habitat quality of the Azorean forests. These indicators provide information about the status and biodiversity dynamics that allow us to support future conservations plans (Tsafack et al. 2023a, Tsafack et al. 2023b).

The LIFE SNAILS project (Support and Naturalisation in Areas of Importance for Land Snails) has the main aim to protect three species of terrestrial molluscs, two snails (*Oxychilusagostinhoi* Martins, 1981 and *Leptaxisminor* Backhuys, 1975) and a semi-slug (*Plutoniaangulosa* (Morelet, 1860)), which are endemic to Santa Maria Island and whose populations are at high risk (two of them assessed as Critically Endangered and one as Endangered on IUCN Red Lists; de Frias Martins (2010), Cameron et al. (2016), de Frias Martins (2022)). The conservation threats for these species primarily include habitat degradation and destruction (e.g. disappearance of their endemic habitat) and, secondarily, the presence of invasive species (particularly *Hedychiumgardnerianum* Sheph. ex Ker Gawl. expansion and exotic tree plantations) and droughts associated with climate change (de Frias Martins 2010, Cameron et al. 2016, de Frias Martins 2022).

## General description

### Purpose

**Figure 1.**
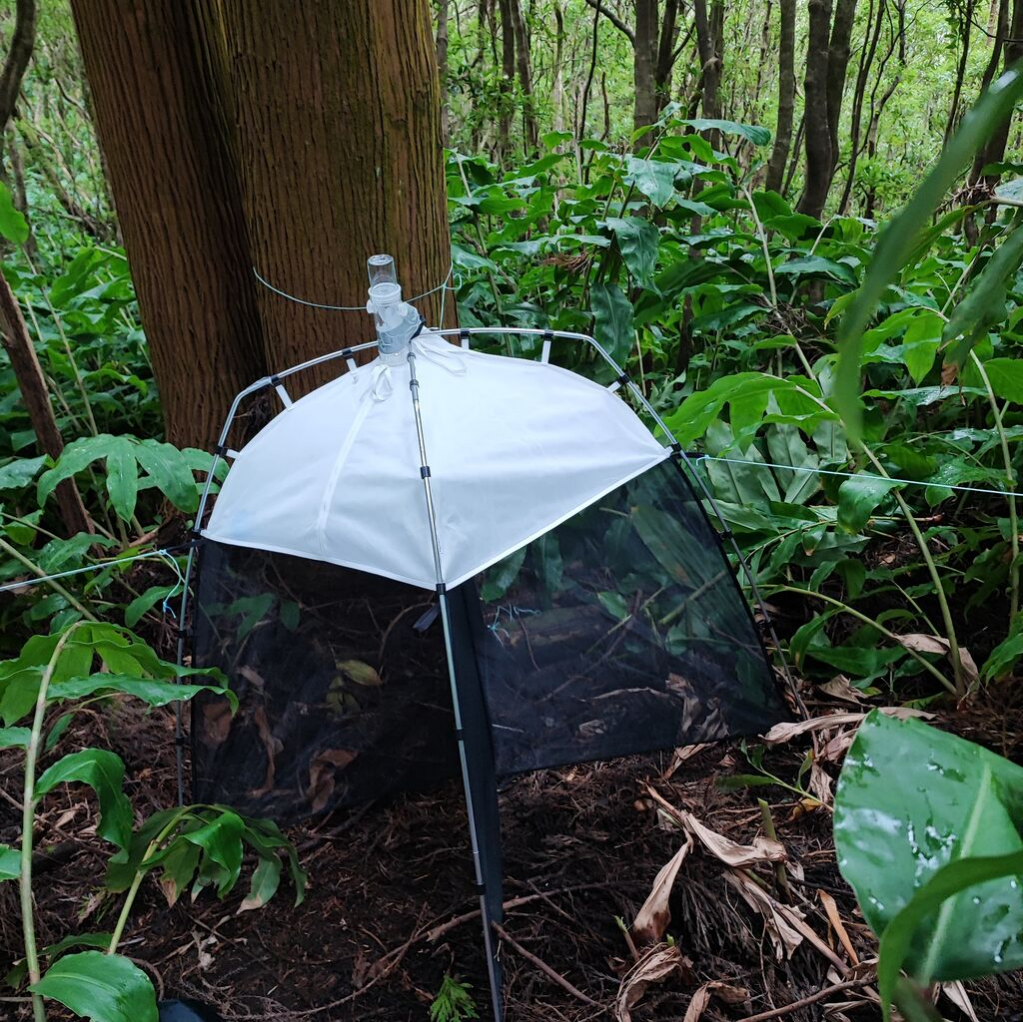
SLAM traps (Sea, Land, Air, Malaise traps) (Credit: Paulo A. V. Borges).

To provide an inventory of arthropods, as principal indicators of habitat quality for endemic and threatened invertebrates, we used SLAM (Sea, Land, Air, Malaise) traps (Fig. 1) deployed on mixed forests of Santa Maria Island, under the scope of the LIFE SNAILS project, which has the main aim of protecting three species of threatened endemic terrestrial molluscs.

### Additional information

The database we present is part of the LIFE SNAILS project (Support and Naturalisation in Areas of Importance for Land Snails), which has the main aim of protecting three species of terrestrial molluscs, two snails (*Oxychilusagostinhoi* and *Leptaxisminor*) and a semi-slug (*Plutoniaangulosa*), endemic to Santa Maria Island and whose populations are threatened (two of them assessed as Critically Endangered and one as Endangered on IUCN Red Lists; de Frias Martins (2010), Cameron et al. (2016), de Frias Martins (2022)).

## Project description

### Title

The use of arthropods as surrogates of habitat quality within the scope of LIFE SNAILS project.

### Personnel

The SLAM monitoring protocol was conceived and led by Paulo A.V. Borges.

Fieldwork (site selection and experimental setting): Nelson B. Moura, Mauro Ponte, Ricardo J.F. Abreu, Paulo A.V. Borges and António Manuel de Frias Martins.

Fieldwork (authorisation): Secretaria Regional do Ambiente e Alterações Climáticas.

Fieldwork: Nelson B. Moura & Paulo A. V. Borges

Parataxonomists: Abrão Leite & Laurine Parmentier.

Taxonomist: Paulo A. V. Borges.

Voucher specimen management: Abrão Leite & Laurine Parmentier.

Database management: Sébastien Lhoumeau and Paulo A. V. Borges.

Darwin Core databases: Sébastien Lhoumeau and Paulo A. V. Borges.

### Study area description

Santa Maria is a small island (area: 97.2 km²; elevation: 590 m a.s.l.) of volcanic origin, belonging to the Oriental group of the Azores Archipelago (36°58′24″N 25°05′40″W). The sampling area consists of mixed-forests of endemic, native and exotic plant species. The main native and endemic species include *Morellafaya* Wilbur, *Ericaazorica* Hochst. ex Seub., *Picconiaazorica* (Tutin) Knobl., *Vacciniumcylindraceum* Sm. or *Laurusazorica* (Seub.) Franco. The exotic species include *Pittosporumundulatum* Vent., *Hedychiumgardnerianum* Sheph. ex Ker Gawl. and also forestry plantations of *Cryptomeriajaponica* (Thunb. ex L.f.) D.Don.

The climate is temperate oceanic, with regular and abundant rainfall, high levels of relative humidity and persistent winds, mainly during the winter and autumn seasons.

### Funding

Secretaria Regional do Ambiente e Alterações Climáticas, Project LIFE SNAILS (LIFE20 NAT/PT/001377).

## Sampling methods

### Study extent

**Figure 2.**
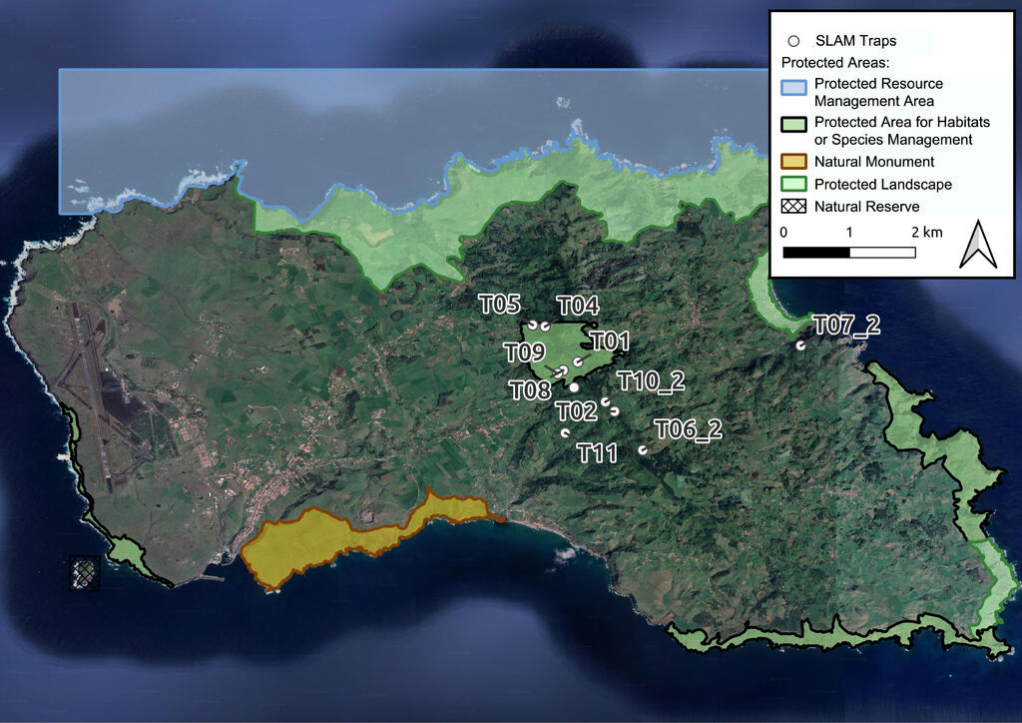
Location of 11 sampled sites on Santa Maria Island (Azores, Portugal). Information about Protected Areas is included.

A total of 11 sites were sampled in Santa Maria Island (Table 1; Fig. 2). The sampling area consisted of mixed-forests of endemic, native and exotic plant species. The main native and endemic species included *Morellafaya*, *Ericaazorica*, *Picconiaazorica*, *Vacciniumcylindraceum* or *Laurusazorica*. The exotic species include *Pittosporumundulatum*, *Hedychiumgardnerianum* and also forestry plantations of *Cryptomeriajaponica*. Information of vegetation composition (dominant plant species in surrounding area) was recorded (see Table 2).

### Sampling description

Passive flight interception SLAM traps (Sea, Land, Air, Malaise traps) (Fig. 1) were used to sample each of 11 selected sites on the mixed-forests of Santa Maria Island, between September and December of 2022.

This trap consists in a structure of 110 x 110 x 110 cm, where the trapped arthropods crawl up the mesh and then fall inside the sampling recipient (Borges et al. 2017). Each one is filled with propylene glycol (pure 1,2-Propanodiol) to kill the captured arthropods and conserve the sample between collections. Although this protocol was developed to sample flying arthropods, by working as an extension of the tree, non-flying species such as spiders can also crawl into the trap (Borges et al. 2017), increasing the range of groups that can be sampled by this technique. As a result of this, previous studies have used these traps to analyse diversity and abundance changes in the arthropod communities in the pristine forests of the Azores (Tsafack et al. 2021, Lhoumeau and Borges 2023, Tsafack et al. 2023a, Tsafack et al. 2023b). The traps were installed during 30 consecutive days in three periods between September and December 2022, after which samples were collected.

Information of vegetation composition (dominant plant species in surrounding area) and elevation were recorded.

### Quality control

All sorted specimens were identified by a taxonomist in the laboratory.

### Step description

A reference collection was made for all collected specimens (whether or not identified at species level) by assigning them a morphospecies code number and depositing them at the Dalberto Teixeira Pombo Insect Collection (DTP), University of Azores (Terceira Island).

## Geographic coverage

### Description

Santa Maria Island, Azores, (Portugal).

### Coordinates

25°5'45.6''S and 36°59'6N Latitude; 25°3'0'W and 36°57'46.8'E' Longitude.

## Taxonomic coverage

### Description

The following Classes and Orders of the Phylum Arthropoda are covered:

**Phylum**: Arthropoda

**Class**: Arachnida, Diplopoda, Insecta

**Order**: Araneae, Opiliones, Pseudoscorpiones, Julida, Archaeognatha, Blattodea, Coleoptera, Dermaptera, Hemiptera, Hymenoptera, Neuroptera, Phasmida, Psocodea, Thysanoptera.

### Taxa included

**Table:** 

Rank	Scientific Name	Common Name
phylum	Arthropoda	Arthropods
class	Arachnida	Arachnids
class	Diplopoda	Millipedes
class	Insecta	Insects

## Temporal coverage

**Data range:** 2022-9-26 – 2022-12-22.

## Collection data

### Collection name

Dalberto Teixeira Pombo Insect Collection

### Collection identifier

DTP

### Specimen preservation method

Ethanol

## Usage licence

### Usage licence

Other

### IP rights notes

Creative Commons Attribution Non-Commercial (CC-BY-NC) 4.0 Licence

## Data resources

### Data package title

Monitoring arthropods under the scope of LIFE-Snails project – Baseline Data

### Resource link

https://doi.org/10.15468/nuue25

### Alternative identifiers

https://www.gbif.org/dataset/715e3b90-a68d-47a5-b676-a8428e1aaf3a; http://ipt.gbif.pt/ipt/resource?r=arthropods_slam_snails&v=1.2

### Number of data sets

2

### Data set 1.

#### Data set name

Event Table

#### Data format

Darwin Core Archive

#### Character set

UTF-8

#### Download URL

http://ipt.gbif.pt/ipt/resource?r=arthropods_slam_snails

#### Data format version

1.2

#### Description

The dataset was published in the Global Biodiversity Information Facility platform, GBIF (Borges et al. 2023). The following data table includes all the records for which a taxonomic identification of the species was possible. The dataset submitted to GBIF is structured as a sample event dataset that has been published as a Darwin Core Archive (DwCA), which is a standardised format for sharing biodiversity data as a set of one or more data tables. The core data file contains 33 records (eventID). This GBIF IPT (Integrated Publishing Toolkit, Version 2.5.6) archives the data and, thus, serves as the data repository. The data and resource metadata are available for download in the Portuguese GBIF Portal IPT (Borges et al. 2023).

**Data set 1.** 

Column label	Column description
eventID	Identifier of the events, unique for the dataset.
stateProvince	Name of the region of the sampling site (Azores).
islandGroup	Name of the archipelago (Azores).
island	Name of the island (Santa Maria).
country	Country of the sampling site (Portugal).
countryCode	ISO code of the country of the sampling site (PT).
municipality	Municipality of the sampling sites (Vila do Porto).
minimumElevationInMetres	The lower limit of the range of elevation (altitude, above sea level), in metres.
decimalLongitude	Approximate centre point decimal longitude of the field site in GPS coordinates.
decimalLatitude	Approximate centre point decimal latitude of the field site in GPS coordinates.
geodeticDatum	The ellipsoid, geodetic datum or spatial reference system (SRS), upon which the geographic coordinates given in decimalLatitude and decimalLongitude are based.
coordinateUncertaintyInMetres	Uncertainty of the coordinates of the centre of the sampling plot.
coordinatePrecision	Precision of the coordinates.
georeferenceSources	A list (concatenated and separated) of maps, gazetteers or other resources used to georeference the Location, described specifically enough to allow anyone in the future to use the same resources.
locationID	Identifier of the location.
locality	Name of the locality.
habitat	The habitat of the sample.
year	Year of the event.
eventDate	Date or date range the record was collected.
sampleSizeValue	The numeric amount of time spent in each sampling.
sampleSizeUnit	The unit of the sample size value.
verbatimEventDate	The verbatim original representation of the date and time information for an Event. In this case, we use the season and year.
samplingProtocol	The sampling protocol used to capture the species (SLAM traps).

### Data set 2.

#### Data set name

Occurrence Table

#### Data format

Darwin Core Archive

#### Character set

UTF-8

#### Download URL

http://ipt.gbif.pt/ipt/resource?r=arthropods_slam_snails

#### Data format version

1.2

#### Description

The dataset was published in the Global Biodiversity Information Facility platform, GBIF (Borges et al. 2023). The following data table includes all the records for which a taxonomic identification of the species was possible. The dataset submitted to GBIF is structured as an occurrence table that has been published as a Darwin Core Archive (DwCA), which is a standard format for sharing biodiversity data as a set of one or more data tables. The core data file contains 578 records (occurrenceID). This GBIF IPT (Integrated Publishing Toolkit, Version 2.5.6) archives the data and, thus, serves as the data repository. The data and resource metadata are available for download in the Portuguese GBIF Portal IPT (Borges et al. 2023).

**Data set 2.** 

Column label	Column description
eventID	Identifier of the events, unique for the dataset.
type	Type of the record, as defined by the Public Core standard.
licence	Reference to the licence under which the record is published.
institutionID	The identity of the institution publishing the data.
collectionID	The identity of the collection publishing the data.
collectionCode	The code of the collection where the specimens are conserved.
institutionCode	The code of the institution publishing the data.
DatasetName	Name of the dataset.
basisOfRecord	The nature of the data record.
recordedBy	A list (concatenated and separated) of names of people, groups or organisations who performed the sampling in the field.
occurrenceID	Identifier of the record, coded as a global unique identifier.
organismQuantity	A number or enumeration value for the quantity of organisms.
organismQuantityType	The type of quantification system used for the quantity of organisms.
sex	The sex and quantity of the individuals captured.
lifeStage	The life stage of the organisms captured.
establishmentMeans	The process of establishment of the species in the location, using a controlled vocabulary: 'native', 'introduced', 'endemic', "indeterminate".
identifiedBy	A list (concatenated and separated) of names of people, groups or organisations who assigned the Taxon to the subject.
dateIdentified	The date on which the subject was determined as representing the Taxon.
scientificName	Complete scientific name including author and year.
kingdom	Kingdom name.
phylum	Phylum name.
class	Class name.
order	Order name.
family	Family name.
genus	Genus name.
specificEpithet	Specific epithet.
infraspecificEpithet	Infraspecific epithet.
taxonRank	Lowest taxonomic rank of the record.
scientificNameAuthorship	Name of the author of the lowest taxon rank included in the record.
identificationRemarks	Information about morphospecies identification (code in Dalberto Teixeira Pombo Collection).

## Additional information

**Results and Discussion**

We collected a total of 3487 individuals, belonging to 118 taxa, three classes, 14 orders and 62 families (Table 3). In general, the most abundant orders were the insect classes of Hemiptera (n = 2218), Psocodea (n = 347) and Coleoptera (n = 335). A total of 94 out of 118 taxa were identified at species or subspecies level, collecting a total of 2284 individuals (= Total value shown in Table 3), where families Cixidae (Hemiptera; n = 938) and Thripidae (Thysanoptera; n = 160) were the most frequently recorded. A total of 1203 individuals were not recorded at species level, most of them belonging to the Aleyrodidae (Hemiptera; n = 914) and Trogiidae (Psocodea; n = 219) families.

**Figure 3.**
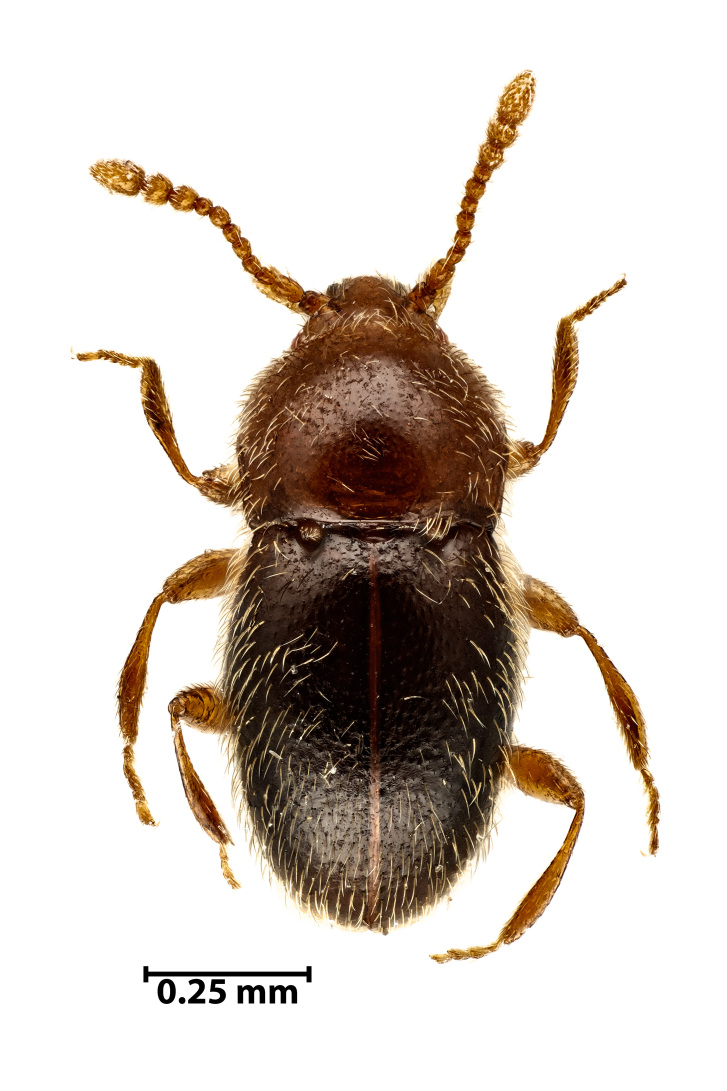
*Cephenniumvalidum* Assing & Meybohm, 2021 (Credit: Javier Torrent).

We registered one new species for the Azores Archipelago, the native beetle *Cephenniumvalidum* Assing & Meybohm, 2021 (Coleoptera, Staphylinidae, Scydmaeninae), recently described from the Iberian Peninsula occurring in northwest Spain and northern Portugal (Assing and Meybohm 2021) (Fig. 3). In addition, we recorded three new species to the Island, the native bug *Piezodoruslituratus* (Fabricius, 1794) (Hemiptera, Pentatomidae), the Azorean endemic beetle *Phloeosinusgillerforsi* Bright, 1987 (Coleoptera, Curculionidae) and the exotic ant *Hypoponerapunctatissima* (Roger, 1859) (Hymenoptera, Formicidae).

In terms of colonisation status, it is remarkable that most of the collected individuals were endemic, adding to a total of 1338 individuals from 21 species, where *Cixiusazomariae* Remane & Asche, 1979 (Hemiptera - Cixiidae; n = 938) and *Pinalitusoromii* J. Ribes, 1992 (Hemiptera - Miridae; n = 126) where the most abundant (Table 3). A total of 504 individuals have been introduced to the islands, belonging to 32 species, where *Hercinothripsbicinctus* (Bagnall, 1919) (Thysanoptera – Thripidae; n = 145) and *Siphantaacuta* (Walker, 1851) (Hemiptera – Flatidae; n = 143) were the most abundant (Table 3). A total of 368 individuals were native non-endemic, belonging to 31 species, where *Zethasimonyi* (Krauss, 1892) (Blattodea – Corydiidae; n = 99) and *Hypoponeraeduardi* (Forel, 1894) (Hymenoptera – Formicidae; n = 41) were the most common species (Table 3). The remaining collected individuals had an indeterminate colonisation status, due to the impossibility for identification at species level or lack of studies on their origin (e.g. *Aleocharabipustulata* (Linnaeus, 1760); Borges et al. (2022)).

According to Index of Biotic Integrity (IBI) proposed by Cardoso et al. (2006) and Tsafack et al. (2023b) we present the IBI Values for each site in Table 4. It is remarkable that all locations inside the protected area have a value of 7, out of a maximum possible value of 14 (Table 4; see Fig. 2). Sites with lower IBI values are associated with parameters related to introduced species, which are more abundant in forests dominated by exotic species and more tolerant to environmental disturbances (Cardoso et al. 2006, Tsafack et al. 2021). On the contrary, higher IBI values are related to a greater abundance of endemic species (e.g. see sites SMR-SNAILS-T06-2 and SMR-NFPA-T01) and correspond to highly pristine and well-conserved areas (Borges et al. 2005).

The study of arthropod communities and the development of monitoring campaigns to study their abundance and species richness have proved to be suitable indicators of habitat quality in the Azores (Cardoso et al. 2006, Tsafack et al. 2023b, Tsafack et al. 2023a). Most of recorded species in mixed forests of Santa Maria were native or endemic (n = 52), including three new records to the Island, as *P.lituratus*, *P.gillerforsi* and *C.validum*, which is an indicator of potential habitat suitability to endemic invertebrates, as the threatened endemic molluscs targeted by the LIFE SNAILS project.

## Figures and Tables

**Table 1.** Details on sites with the decimal longitude and latitude and minimum elevation in metres (m).

**Site Code**	**Locality**	**Longitude**	**Latitude**	**Elevation (m)**
SMR-NFPA-T01	Pico Alto T01	-25.087560	36.978040	460
SMR-SNAILS-T02	Casa dos Picos	-25.081848	36.969886	400
SMR-SNAILS-T03	Linha Média Tensão	-25.088924	36.973938	374
SMR-SNAILS-T04	Trilho BTT_Alto Nascente_1	-25.093743	36.984282	443
SMR-SNAILS-T05	Trilho BTT_Alto Nascente_2	-25.095865	36.984520	400
SMR-SNAILS-T06-2	Fontinhas Florestal Miradouro	-25.077136	36.963267	400
SMR-SNAILS-T07-2	Ribeira do Salto	-25.050221	36.981027	208
SMR-SNAILS-T08	Trilho Areia Branca 1	-25.090618	36.976793	419
SMR-SNAILS-T09	Trilho Areia Branca 2	-25.091595	36.976312	397
SMR-SNAILS-T10-2	Piquinhos	-25.083498	36.971465	423
SMR-SNAILS-T11	Ribeira da Fonte Rainha	-25.090315	36.966181	188

**Table 2.** Details on the plant species in each site.

**Site Code**	**Locality**	**Main Plant Composition**
SMR-NFPA-T01	Pico Alto T01	Endemic plants: *Picconiaazorica*, *Ericaazorica*, *Laurusazorica*, *Vacciniumcylindraceum* Native plants: *Morellafaya*, *Myrsineretusa* Exotic invasive plants: *Pittosporumundulatum*, *Hedychiumgardnerianum* High diversity of endemic and native Pteridophyta and Bryophyta
SMR-SNAILS-T02	Casa dos Picos	Exotic invasive plants: *Pittosporumundulatum*, *Hedychiumgardnerianum*, *Acacia* spp.
SMR-SNAILS-T03	Linha Média Tensão	Endemic plants: *Picconiaazorica* Exotic invasive plants: *Cryptomeriajaponica*, *Pittosporumundulatum*, *Hedychiumgardnerianum*, *Rubusulmifolius* High diversity of endemic and native Pteridophyta and Bryophyta
SMR-SNAILS-T04	Trilho BTT_Alto Nascente_1	Endemic plants: *Picconiaazorica*. Exotic invasive plants: *Cryptomeriajaponica*, *Pittosporumundulatum*, *Hedychiumgardnerianum*, *Pisidium* sp. High diversity of endemic and native Pteridophyta and Bryophyta
SMR-SNAILS-T05	Trilho BTT_Alto Nascente_2	Exotic invasive plants: *Eucalyptus* spp., *Pittosporumundulatum*, *Hedychiumgardnerianum*, *Pisidium* sp. High diversity of endemic and native Pteridophyta and Bryophyta
SMR-SNAILS-T06-2	Fontinhas Florestal Miradouro	Endemic plants: *Picconiaazorica*, *Laurusazorica*. Exotic invasive plants: *Cryptomeriajaponica*, *Pittosporumundulatum*, *Hedychiumgardnerianum*, *Rubusulmifolius*
SMR-SNAILS-T07-2	Ribeira do Salto	Endemic plants: *Picconiaazorica* Exotic invasive plants: *Pittosporumundulatum*, *Hedychiumgardnerianum* High diversity of endemic and native Pteridophyta and Bryophyta
SMR-SNAILS-T08	Trilho Areia Branca 1	Exotic invasive plants: *Pittosporumundulatum*, *Hedychiumgardnerianum* High diversity of endemic and native Pteridophyta and Bryophyta
SMR-SNAILS-T09	Trilho Areia Branca 2	Exotic invasive plants: *Cryptomeriajaponica*, *Hedychiumgardnerianum*
SMR-SNAILS-T10-2	Piquinhos	Endemic plants: *Picconiaazorica*, *Ericaazorica* Exotic invasive plants: *Pittosporumundulatum*, *Hedychiumgardnerianum* High diversity of endemic and native Pteridophyta and Bryophyta
SMR-SNAILS-T11	Ribeira da Fonte Rainha	Exotic invasive plants: *Pittosporumundulatum*, *Hedychiumgardnerianum*

**Table 3.** Inventory of arthropod species collected between September and December of 2022, on mixed-forests of Santa Maria Island (Azores), including order and family names, colonisation status (CS) (END - endemic from the Azores; NAT - native non-endemic; INT - introduced species; IND - indeterminate origin) (Borges et al. 2022) and overall abundance data (N) (adults plus juveniles). Individuals that were not identified to the species level have been excluded from this table.

**Class**	**Order**	**Family**	**Species**	**CS**	**N**
Arachnida	Araneae	Araneidae	*Gibbaraneaoccidentalis* Wunderlich, 1989	END	24
Arachnida	Araneae	Araneidae	*Mangoraacalypha* (Walckenaer, 1802)	INT	1
Arachnida	Araneae	Cheiracanthiidae	*Cheiracanthiumerraticum* (Walckenaer, 1802)	INT	5
Arachnida	Araneae	Cheiracanthiidae	*Cheiracanthiummildei* L. Koch, 1864	INT	8
Arachnida	Araneae	Clubionidae	*Clubionaterrestris* Westring, 1851	INT	1
Arachnida	Araneae	Clubionidae	*Porrhoclubionadecora* (Blackwall, 1859)	NAT	16
Arachnida	Araneae	Dictynidae	*Lathysdentichelis* (Simon, 1883)	NAT	6
Arachnida	Araneae	Dysderidae	*Dysderacrocata* C. L. Koch, 1838	INT	4
Arachnida	Araneae	Linyphiidae	*Acorigoneacoreensis* (Wunderlich, 1992)	END	1
Arachnida	Araneae	Linyphiidae	*Osteariusmelanopygius* (O. Pickard-Cambridge, 1880)	INT	2
Arachnida	Araneae	Linyphiidae	*Savigniorrhipisacoreensis* Wunderlich, 1992	END	17
Arachnida	Araneae	Linyphiidae	*Tenuiphantesmiguelensis* (Wunderlich, 1992)	NAT	5
Arachnida	Araneae	Mimetidae	*Erofurcata* (Villers, 1789)	INT	1
Arachnida	Araneae	Salticidae	*Macaroeriscata* (Blackwall, 1867)	NAT	1
Arachnida	Araneae	Salticidae	*Neonacoreensis* Wunderlich, 2008	END	1
Arachnida	Araneae	Segestriidae	*Segestriaflorentina* (Rossi, 1790)	INT	3
Arachnida	Araneae	Tetragnathidae	*Leucognathaacoreensis* Wunderlich, 1992	END	13
Arachnida	Araneae	Theridiidae	*Cryptachaeablattea* (Urquhart, 1886)	INT	2
Arachnida	Araneae	Theridiidae	*Lasaeolaoceanica* Simon, 1883	END	5
Arachnida	Araneae	Theridiidae	*Rugathodesacoreensis* Wunderlich, 1992	END	11
Arachnida	Araneae	Theridiidae	*Steatodanobilis* (Thorell, 1875)	NAT	1
Arachnida	Opiliones	Leiobunidae	*Leiobunumblackwalli* Meade, 1861	NAT	28
Arachnida	Pseudoscorpiones	Chthoniidae	*Chthoniusischnocheles* (Hermann, 1804)	INT	1
Diplopoda	Julida	Julidae	*Ommatoiulusmoreleti* (Lucas, 1860)	INT	5
Insecta	Archaeognatha	Machilidae	*Diltasaxicola* (Womersley, 1930)	NAT	1
Insecta	Blattodea	Corydiidae	*Zethasimonyi* (Krauss, 1892)	NAT	99
Insecta	Coleoptera	Apionidae	*Aspidapionradiolus* (Marsham, 1802)	INT	6
Insecta	Coleoptera	Chrysomelidae	*Chaetocnemahortensis* (Fourcroy, 1785)	INT	1
Insecta	Coleoptera	Chrysomelidae	*Epitrixcucumeris* (Harris, 1851)	INT	3
Insecta	Coleoptera	Chrysomelidae	*Longitarsuskutscherai* (Rye, 1872)	INT	24
Insecta	Coleoptera	Corylophidae	*Sericoderuslateralis* (Gyllenhal, 1827)	INT	1
Insecta	Coleoptera	Curculionidae	*Calacallessubcarinatus* (Israelson, 1984)	END	17
Insecta	Coleoptera	Curculionidae	*Cathormioceruscurvipes* (Wollaston, 1854)	NAT	1
Insecta	Coleoptera	Curculionidae	*Charagmusgressorius* (Fabricius, 1792)	NAT	2
Insecta	Coleoptera	Curculionidae	*Mecinuspascuorum* (Gyllenhal, 1813)	INT	1
Insecta	Coleoptera	Curculionidae	*Mogulonesgeographicus* (Goeze, 1777)	INT	1
Insecta	Coleoptera	Curculionidae	*Phloeosinusgillerforsi* Bright, 1987	END	1
Insecta	Coleoptera	Curculionidae	*Rhopalomesitestardyi* (Curtis, 1825)	INT	1
Insecta	Coleoptera	Curculionidae	*Sitonadiscoideus* Gyllenhal, 1834	INT	2
Insecta	Coleoptera	Elateridae	*Heteroderesazoricus* (Tarnier, 1860)	END	47
Insecta	Coleoptera	Leiodidae	*Catopscoracinus* Kellner, 1846	NAT	14
Insecta	Coleoptera	Nitidulidae	*Stelidotageminata* (Say, 1825)	INT	20
Insecta	Coleoptera	Phalacridae	*Stilbustestaceus* (Panzer, 1797)	NAT	2
Insecta	Coleoptera	Ptiliidae	*Ptenidiumpusillum* (Gyllenhal, 1808)	INT	18
Insecta	Coleoptera	Staphylinidae	*Cephenniumvalidum* Assing & Meybohm, 2021	NAT	1
Insecta	Coleoptera	Silvanidae	*Cryptamorphadesjardinsii* (Guérin-Méneville, 1844)	INT	3
Insecta	Coleoptera	Staphylinidae	*Aleocharabipustulata* (Linnaeus, 1760)	IND	2
Insecta	Coleoptera	Staphylinidae	*Athetaaeneicollis* (Sharp, 1869)	IND	11
Insecta	Coleoptera	Staphylinidae	*Athetafungi* (Gravenhorst, 1806)	IND	3
Insecta	Coleoptera	Staphylinidae	*Carpelimuscorticinus* (Gravenhorst, 1806)	IND	2
Insecta	Coleoptera	Staphylinidae	*Cordaliaobscura* (Gravenhorst, 1802)	IND	2
Insecta	Coleoptera	Staphylinidae	*Euconnusazoricus* Franz, 1969	END	1
Insecta	Coleoptera	Staphylinidae	*Notothectadryochares* (Israelson, 1985)	END	37
Insecta	Coleoptera	Staphylinidae	*Phloeonomuspunctipennis* Thomson, 1867	IND	1
Insecta	Coleoptera	Staphylinidae	*Proteinusatomarius* Erichson, 1840	IND	2
Insecta	Coleoptera	Staphylinidae	*Tachyporuschrysomelinus* (Linnaeus, 1758)	IND	7
Insecta	Coleoptera	Staphylinidae	*Tachyporusnitidulus* (Fabricius, 1781)	IND	43
Insecta	Coleoptera	Tenebrionidae	*Lagriahirta* (Linnaeus, 1758)	INT	4
Insecta	Coleoptera	Zopheridae	*Tarphiusrufonodulosus* Israelson, 1984	END	6
Insecta	Dermaptera	Forficulidae	*Forficulaauricularia* Linnaeus, 1758	INT	71
Insecta	Hemiptera	Cicadellidae	*Eupteryxazorica* Ribaut, 1941	END	15
Insecta	Hemiptera	Cicadellidae	*Eupteryxfilicum* (Newman, 1853)	NAT	3
Insecta	Hemiptera	Cixiidae	*Cixiusazomariae* Remane & Asche, 1979	END	938
Insecta	Hemiptera	Delphacidae	*Kelisiaribauti* Wagner, 1938	NAT	1
Insecta	Hemiptera	Flatidae	*Cyphopterumadscendens* (Herrich-Schäffer, 1835)	NAT	13
Insecta	Hemiptera	Flatidae	*Siphantaacuta* (Walker, 1851)	INT	143
Insecta	Hemiptera	Liviidae	*Strophingiaharteni* Hodkinson, 1981	END	16
Insecta	Hemiptera	Lygaeidae	*Kleidocerysericae* (Horváth, 1909)	NAT	6
Insecta	Hemiptera	Lygaeidae	*Nysiusatlantidum* Horváth, 1890	END	1
Insecta	Hemiptera	Microphysidae	*Loriculacoleoptrata* (Fallén, 1807)	NAT	4
Insecta	Hemiptera	Miridae	*Monalocorisfilicis* (Linnaeus, 1758)	NAT	2
Insecta	Hemiptera	Miridae	*Pinalitusoromii* J. Ribes, 1992	END	126
Insecta	Hemiptera	Nabidae	*Nabispseudoferusibericus* Remane, 1962	NAT	4
Insecta	Hemiptera	Pentatomidae	*Nezaraviridula* (Linnaeus, 1758)	INT	2
Insecta	Hemiptera	Pentatomidae	*Piezodoruslituratus* (Fabricius, 1794)	NAT	1
Insecta	Hemiptera	Rhyparochromidae	*Scolopostethusdecoratus* (Hahn, 1833)	NAT	10
Insecta	Hemiptera	Triozidae	*Triozalaurisilvae* Hodkinson, 1990	NAT	4
Insecta	Hymenoptera	Formicidae	*Hypoponeraeduardi* (Forel, 1894)	NAT	41
Insecta	Hymenoptera	Formicidae	*Hypoponerapunctatissima* (Roger, 1859)	INT	2
Insecta	Hymenoptera	Formicidae	*Lasiusgrandis* Forel, 1909	NAT	16
Insecta	Neuroptera	Hemerobiidae	*Hemerobiusazoricus* Tjeder, 1948	END	3
Insecta	Phasmida	Phasmatidae	*Carausiusmorosus* (Sinéty, 1901)	INT	1
Insecta	Psocodea	Caeciliusidae	*Valenzuelaburmeisteri* (Brauer, 1876)	NAT	18
Insecta	Psocodea	Caeciliusidae	*Valenzuelaflavidus* (Stephens, 1836)	NAT	12
Insecta	Psocodea	Ectopsocidae	*Ectopsocusbriggsi* McLachlan, 1899	INT	10
Insecta	Psocodea	Elipsocidae	*Elipsocusazoricus* Meinander, 1975	END	52
Insecta	Psocodea	Elipsocidae	*Elipsocusbrincki* Badonnel, 1963	END	6
Insecta	Psocodea	Epipsocidae	*Bertkauialucifuga* (Rambur, 1842)	NAT	5
Insecta	Psocodea	Psocidae	*Atlantopsocusadustus* (Hagen, 1865)	NAT	8
Insecta	Psocodea	Trichopsocidae	*Trichopsocusclarus* (Banks, 1908)	NAT	16
Insecta	Thysanoptera	Phlaeothripidae	*Hoplothripscorticis* (De Geer, 1773)	NAT	25
Insecta	Thysanoptera	Thripidae	*Ceratothripsericae* (Haliday, 1836)	NAT	3
Insecta	Thysanoptera	Thripidae	*Heliothripshaemorrhoidalis* (Bouché, 1833)	INT	12
Insecta	Thysanoptera	Thripidae	*Hercinothripsbicinctus* (Bagnall, 1919)	INT	145

**Table 4.** Values of Index of Biotic Integrity (IBI) in a scale of 0 to 14 points, for Autumn 2022, also indicating the values for each of the months studied (October, November and December 2022), for each sampled site in mixed forests of Santa Maria Island.

Site Code	IBI - Autumn	IBI - October	IBI - November	IBI - December
SMR-NFPA-T01	7	8	8	10
SMR-SNAILS-T02	10	7	6	10
SMR-SNAILS-T03	6	9	8	6
SMR-SNAILS-T04	7	10	5	8
SMR-SNAILS-T05	7	7	7	7
SMR-SNAILS-T06-2	10	11	8	9
SMR-SNAILS-T07-2	7	8	5	6
SMR-SNAILS-T08	7	8	5	6
SMR-SNAILS-T09	7	7	6	8
SMR-SNAILS-T10-2	4	7	5	6
SMR-SNAILS-T11	5	8	6	4
